# Temporomandibular disorders and psychosocial status in osteogenesis imperfecta - a cross-sectional study

**DOI:** 10.1186/s12903-018-0497-3

**Published:** 2018-03-07

**Authors:** K. H. Bendixen, H. Gjørup, L. Baad-Hansen, J. Dahl Hald, T. Harsløf, M. H. Schmidt, B. L. Langdahl, D. Haubek

**Affiliations:** 1 Section of Orofacial Pain and Jaw Function, Department of Dentistry and Oral Health, Aarhus University, 8000 Aarhus, DK Denmark; 2 Center for Oral Health in Rare Diseases, Department of Maxillofacial Surgery, Aarhus University Hospital, Aarhus, Denmark; 3 Department of Endocrinology and Internal Medicine, Aarhus University Hospital, Aarhus, Denmark; 4 Aarhus Municipality, Aarhus, Denmark; 5 Section for Pediatric Dentistry, Department of Dentistry and Oral Health, Aarhus University, Aarhus, Denmark

**Keywords:** Rare diseases, Heritable connective tissue disorders, Bone fractures, Dental anomalies, Pain, Jaw function

## Abstract

**Background:**

Osteogenesis Imperfecta (OI) is characterized by a number of deviations in the orofacial region. The aims of the present study were to investigate the occurrence of temporomandibular disorders, to evaluate the psychosocial status, and to assess the dental occlusion in a population of adult OI patients.

**Methods:**

Participants (*n* = 75) were classified with mild OI, type I (*n* = 56), or moderate-severe OI, type III and IV (*n* = 19). OI patients were examined according to the Research Diagnostic Criteria for Temporomandibular Disorders (axis I and II).

**Results:**

Temporomandibular disorders and functional limitations in the orofacial region were rare and did not differ between patients with mild and moderate-severe OI (*P* > 0.050). No significant differences between Graded Chronic Pain Scale grades 0, 1, and 2 were found in mild OI vs. moderate-severe OI (*P* > 0.160). Few patients (16%) had signs of depression, but close to half (48%) had signs of somatization. Patients with moderate-severe OI had a lower mean number of teeth compared to patients with mild OI (*P* <  0.050). In general, malocclusions were prevalent, and mandibular overjet and posterior cross-bite were found more often in moderate-severe OI compared with mild (*P* <  0.050).

**Conclusions:**

Patients with moderate-severe OI had more malocclusions than patients with mild OI. The psychosocial status of OI patients was remarkably healthy considering the severity of this disabling systemic disorder. The bodily pain complaints frequently reported in OI patients were not largely reflected in the orofacial area as painful temporomandibular disorders.

## Background

Osteogenesis imperfecta (OI) is a rare, heritable connective tissue disorder, characterized by fragile bones, which results in an increased risk of fractures from low energy trauma [1]. Growth retardation, blue sclerae, hearing loss, and disturbances in the dental development also occur. OI is caused by defective collagen type 1 synthesis, which constitutes an essential part of the connective tissue. The prevalence of OI is estimated to be 11 per 100.000 [2]. The most frequently used classification comprises four clinical subtypes (I-IV): OI type I (mild and no bone deformities), OI type II (severe bone deformities and perinatal death), OI type III (manifest growth retardation and severely progressing bone deformities), and OI type IV (mild growth retardation and moderate bone deformities) [3]. The classical dental aberration of OI is dentinogenesis imperfecta (DI). The clinical characteristics of DI are grayish or brownish discoloration of the dentition, obliteration of the dental cavum, shortness of the roots, and cervical constriction [4, 5]. The prevalence of DI in OI populations is 19–42% [6, 7]. Furthermore, Class III malocclusion with mandibular overjet is prevalent in OI patients, especially OI type III [8, 9]. Pain in relation to the skeleton and certain physical disabilities are frequent in OI patients [10–13].

Temporomandibular Disorders (TMD) consist of a heterogeneous collection of conditions characterized by pain and/or functional limitations in the masticatory muscles, the temporomandibular joints, and associated tissues [14–17]. The TMD major subtypes according to the Research Diagnostic Criteria for Temporomandibular Disorders (RDC/TMD) are myofascial pain, disc displacements, joint pain, and degenerative and inflammatory joint disease [14]. The estimated prevalence of TMD in the general population is 5–12% [15, 18]. TMD is considered to be the most common orofacial pain condition of non-dental origin, and are the second most frequently occurring musculoskeletal condition, resulting in pain and functional impairment [19]. Female/male ratio is approximately 2:1 [15]. The majority of TMD patients suffer from myofascial pain [20, 21]. By means of the RDC/TMD, both the axis I, the physical diagnoses and the axis II, the psychosocial status can be evaluated [22].

According to our knowledge, studies on pain and functional limitations related to temporomandibular disorders in OI patients are absent. Taken the disabling impacts of some OI types in account, we hypothesize that these circumstances are also reflected in the orofacial area.

The aims of the present study were: i) to report on the occurrence of temporomandibular disorders (axis I), ii) to assess jaw function, iii) to evaluate the psychosocial status (axis II), and iiii) to assess the dental occlusion in adult OI patients.

## Methods

### Study population

The present study was conducted as a part of a cross-sectional study investigating adult Danish patients with OI [23]. The patients were identified by search in medical files of the university hospitals in Denmark, by contact to doctors at regional hospitals, and by contact to the Danish Osteogenesis Imperfecta Society. Ninety-one individuals gave their consent to participate in the main study, and the OI diagnosis was confirmed in 85 of these individuals. The 85 participants were classified into OI types I, III, and IV [3] and asked to participate in the study investigating orofacial health. A total of 75 patients accepted the invitation (OI type I: *n* = 56, type III: *n* = 7, and type IV: *n* = 12). The ten persons who declined to participate were OI type I: *n* = 4, type III: *n* = 4, and type IV: *n* = 2. The RDC/TMD examinations were carried out at the Department of Dentistry and Oral Health, Health, Aarhus University, Denmark in the years 2011 to 2012. Thus, a total of 75 OI patients (53.3% females and 46.7% males, the mean age 45.5 yrs., SD = 14.7, range: 20 to 77) were enrolled in the present study and subsequently grouped into mild OI (OI type I) and moderate-severe OI (OI type III and IV).

### Study design

The study was conducted in accordance with the guidelines of the Helsinki Declaration and approved by the Central Denmark Region Committees on Biomedical Research Ethics (M-20100108). The study conforms to STROBE Guidelines.

The temporomandibular function of the OI patients was assessed in accordance with the guidelines of the RDC/TMD, in relation to both axis I and II [14]. One of three involved examiners (MHS, HG, and RF) performed the RDC/TMD clinical examination. An experienced user of the RDC/TMD clinical examination method (LBH) trained the three examiners. All 75 patients underwent the RDC/TMD examination (axis I) and filled out the RDC/TMD History Questionnaire (axis II) for the assessment of the psychosocial status [14, 22].

The presence of temporomandibular disorders was assessed in relation to OI subtypes and categorized in Group I: Muscle Disorders (Myofascial pain (Ia), Myofascial pain with limited opening (Ib), and No Group (Idx) (which is no group I diagnosis)); Group II: Disc Displacements (Disc displacement with reduction (IIa), Disc displacement without reduction with limited opening (IIb), Disc displacement without reduction without limited opening (IIc), and No Group II Diagnosis), and Group III: Other joint conditions (Arthralgia (IIIa), Osteoarthritis (IIIb), Osteoarthrosis (IIIc), and No Group III Diagnosis).

Psychosocial status were assessed by means of the Graded Chronic Pain Scale (GCPS) [24] to rate the level of the severity of chronic pain and by the use of the Symptom Checklist 90-R (SCL-90-R) for the evaluation of depression (DEP) and somatization (i.e., non-specific physical symptoms, pain items included) (SOM) levels [25]. The GCPS, which is a valid instrument based on self-reporting, consists of six items assessed on a 10-point scales. It categorizes patients according to severity in five levels of chronic pain grades: 0 = No disability, 1 = Low disability and low pain intensity, 2 = Low disability and high pain intensity, 3 = High disability and moderately limiting, and 4 = High disability and severely limiting [24, 26]. DEP and SOM levels were assessed by the use of a 20-item instrument. Both the DEP and the SOM scores were calculated and categorized according to severity. DEP: < 0.535 = normal, 0.535–1.105 = indication of moderate depression, and > 1.105 = the presence of severe depressive symptoms. SOM: < 0.5 = normal, 0.5–1.0 = indication of moderate somatization, and > 1.0 = the presence of severe somatization symptoms [25, 26].

**Fig. 1:**
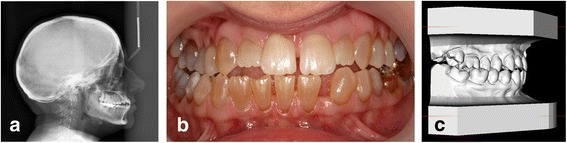
Patient Case - OI Type IV. **a** Lateral Cephalogram. **b** Clinical Photo – Frontal Aspect in Maximum Occlusion. **c** Digital Study Models Obtained by O3DM® (Ortolab, Częstochowa, Poland)

The dental occlusion was evaluated on the digital study models and from the clinical photos and performed by an experienced orthodontist (HG) (Fig. 1). The evaluation of occlusion included any fixed dental prosthesis, but not removable dentures. The horizontal overjet (HO) was evaluated as normal (0 mm < HO < 6 mm), reduced (HO ≤ 0 mm), or increased (HO ≥ 6 mm) according to a modification of methods by Bjørk and coworkers [27]. Reduced HO was characterized as mandibular overbite (MOB). The anterior occlusion was characterized as open (frontal open bite), if the upper incisors did not occlude with the lower dentition.

Non-occlusion posteriorly was defined as the absence of occlusion between premolars and molars in the same side. The non-occlusion had to be present either in one or in both sides. Non-occlusion, because of missing teeth, was included. Posterior cross-bite was defined as the presence of cross-bite on at least two teeth (premolars or molars) in the same side. The posterior cross-bite had to be present either in one or in both sides. If both premolars and molars of the same side were absent in the same jaw, registration of that side was omitted.

### Statistical analyses

Descriptive statistics were used to summarize all data. The frequencies of the temporomandibular disorders diagnoses, the GCPS, DEP, and SOM scores are presented in %. All differences, including the number of teeth and the presence of malocclusion according to OI type (mild or moderate-severe), were evaluated by Students *t*-test or Fisher’s Exact Test. Prior to analyses data were tested for normality by the use of QQ-plots. Data are presented as mean ± standard deviation (SD). Values of *P* < 0.050 were considered statistically significant.

## Results

### Temporomandibular disorders (axis I)

The Group I disorders, “Myofascial pain” (Ia) and “Myofascial pain with limited opening” (Ib), were rare in the mild as well as in the moderate-severe OI type as reported in Table 1, and no differences between the groups were found (*P* > 0.999).

**Table 1:** Temporomandibular Disorders Prevalence According to the Research Diagnostic Criteria for Temporomandibular Disorders (RDC/TMD) Related to Osteogenesis Imperfacta (OI) Subtypes

		OI TYPE	
		Mild (*n* = 56)	Moderate-severe (*n* = 19)
Group I			
1	Myofascial pain (Ia)	3 (5.4%)	1 (5.3%)
2	Myofascial pain with limited opening (Ib)	2 (3.6%)	0
0	No group (Idx)	51 (91.1%)	18 (94.7%)
Group II			
1	Disc displacement with reduction (IIa)	10 (18.2%)	1 (5.6%)
2	Disc displacement without reduction with limited opening (IIb)	0	0
3	Disc displacement without reduction without limited opening (IIc)	0	0
0	No group II Diagnosis	45 (81.8%)	17 (94.4%)
Group III			
1	Arthralgia (IIIa)	3 (5.7%)	0
2	Osteoarthritis (IIIb)	2 (3.8%)	0
3	Osteoarthrosis (IIIc)	4 (7.5%)	2 (11.1%)
0	No group III Diagnosis	44 (83.0%)	16 (88.9%)

Mild: OI type 1. Moderate-severe: OI type 3 and 4. Group I: Muscle Conditions. Group II: Disc Displacements. Group III: Other Joint Conditions. Group II and III diagnoses: Either present in one or both joints. Missing data: Group I: None. Group II: One mild type and one moderate-severe type. Group III: Three mild types and one moderate-severe type

The proportion of the Group II disorder “Disc displacement with reduction” (IIa) did not differ between the groups (*P* = 0.273) (Table 1). The two remaining Group II disorders “Disc displacement without reduction with limited opening” (IIb) or “Disc displacement without reduction without limited opening” (IIc) were not present in the study population (Table 1).

The Group III disorders, including “Arthralgia” (IIIa), “Osteoarthritis” (IIIb), and “Osteoarthrosis” (IIIc), were rare, and there were no differences between the groups (*P* > 0.566) (Table 1). Group II and III diagnoses were recorded if present in one or in both joints.

In total, 7 patients, all of whom were mild OI type, had more than one TMD diagnosis.

### Functional status

The mean value of the maximum unassisted opening capacity was 50.6 mm ± 8.1 mm in the mild OI type, ranging from 32 mm to 70 mm, and 46.1 mm ± 7.9 mm in moderate-severe OI, ranging from 35 mm to 58 mm (*P* = 0.055) (Table 2). Reduced jaw opening capacity (< 40 mm including vertical overlap) was seen in 8.9% of the patients with mild OI and in 25.0% of patients with moderate-severe OI (*P* = 0.037) (Table 2).

**Table 2:** Prevalence of Jaw Function and Limitations Related to Osteogenesis Imperfecta (OI) Subtypes

	OI TYPE	
	Mild (*n* = 56)	Moderate-severe (*n* = 19)
Function		
Maximum unassisted opening (mm) (mean ± SD)	50.6 ± 8.1	46.1 ± 7.9^a^
Reduced jaw opening capacity (%)	8.9	25.0^*^
Limitations (%)		
Chewing	6.7^b^	16.7^a^
Drinking	0.0^b^	0.0^a^
Exercising	0.0^b^	0.0^a^
Eating hard foods	21.7^b^	44.4^a^
Eating soft foods	0.0^b^	0.0^a^
Smiling/laughing	4.4^b^	5.5^a^
Sexual activity	2.2^b^	5.5^a^
Cleaning teeth or face	6.7^b^	0.0^a^
Yawning	8.9^b^	5.5^a^
Swallowing	2.2^b^	0.0^a^
Talking	0.0^b^	0.0^a^
Having the usual facial appearance	0.0^b^	0.0^a^

Mild: OI type 1. Moderate-severe: OI type 3 and 4

Missing data: ^a^ *n* = 1–3; ^b^ *n* = 10–12

^*^P = 0.037, Fischer’s Exact Test

Functional limitations (Question 19) were detected to a minor extent, apart from chewing (6.7% in mild and 16.7% in moderate-severe OI) (*P* = 0.340) and eating hard foods (21.7% in mild and 44.4% in moderate-severe OI type) (*P* = 0.119) (Table 2). All other potential limitations were present below 9%, and no differences between groups were found (*P* > 0.493).

### Psychosocial status (axis II)

Scores from the GCPS revealed Grade 0, which is no TMD pain in the prior six months, in 44 of the 56 patients (78.6%) with mild OI, and in 18 of the 19 (94.8%) with moderate-severe OI (Table 3). There was no significant difference between the groups (*P* = 0.161). Grade 1 (low disability and low pain intensity) was found in 10 of the 56 (17.9%) patients with mild OI and in one of the 19 (5.6%) patients with moderate-severe OI, but the difference between the groups was not significant (*P* = 0.272). Grade 2 (low disability and high pain intensity) was present in two patients (3.6%) with mild OI, but not present in any of the patients with moderate-severe OI. There was no difference between the groups (*P* > 0.999). Grade 3 (high disability and moderately limiting) and grade 4 (high disability and severely limiting) were not found in any OI patients (Table 3).

**Table 3:** Psycosocial Status - Prevalence of RDC/TMD Axis II Findings According to OI Type

		OI TYPE	
		Mild (*n* = 56)	Moderate-severe (*n* = 19)
Chronic Pain Grade Classification	Grade 0	44 (78.6%)	18 (94.8%)
	Grade 1	10 (17.9%)	1 (5.6%)
	Grade 2	2 (3.6%)	0
	Grade 3	0	0
	Grade 4	0	0
Depression	Normal	37 (84.1%)	12 (85.8%)
	Moderate	3 (6.8%)	2 (14.3%)
	Severe	4 (9.1%)	0
Somatization	Normal	26 (53.1%)	9 (50.0%)
	Moderate	16 (32.7%)	4 (22.2%)
	Severe	7 (14.3%)	5 (27.8%)

Grade 0: No TMD pain in the prior 6 months. Grade 1: Low disability - Low Intensity = CPI < 50, and less than 3 DP. Grade 2: Low Disability - High Intensity = CPI ≥ 50, and less than 3 DP. Grade 3: High Disability - Moderately Limiting = 3 to 4 DP, regardless of CPI. Grade 4: High Disability - Severely Limiting = 5 to 6 DP, regardless of CPI. CPI: Characteristic Pain Intensity. DP: Disability Points

Depression: Missing data: Mild OI: *n* = 12; moderate-severe OI: *n* = 5. Normal: < 0.535; Moderate: 0.535–1.105; Severe: < 1.105

Somatization: Missing data mild OI: *n* = 7; moderate-severe OI: *n* = 1. Normal: < 0.5; Moderate: 0.5–1.0; Severe: > 1.0

DEP scores were obtained from 78.6% of the patients with mild OI and from 73.7% of the patients with moderate-severe OI as reported in Table 3. Data from 12 patients with mild OI and 5 with moderate-severe OI were missing, i.e., if the questionnaire was not correctly filled out. No significant differences between groups in any of the DEP score levels were found (*P* = 0.564). Likewise, SOM scores were obtained from 87.5% of the patients with mild OI and from 94.7% of the patients with moderate-severe OI as reported in Table 3. Data from seven patients with mild OI type and one patient with moderate-severe OI were missing. No significant differences between groups in any of the SOM score levels were found (*P* = 0.282).

### Occlusion

Registration of the occlusion was omitted in six out of 75 patients because of missing teeth or missing data. Three patients (OI type I) had a full denture in the edentulous upper jaw. Two patients (one OI type I and one OI type III) had extensive loss of teeth in both the upper and lower jaw. In addition, neither clinical photos nor study cast were obtained in one patient (OI type III). The mean number of teeth was significantly reduced in patients with moderate-severe OI compared to patients with mild OI (*P* < 0.050) (Table 4). However, the three patients with full denture in the upper jaw and one patient with unspecified tooth loos all had mild OI, and the range of tooth number was greatest in the group with mild OI (Table 4). Malocclusion, in terms of mandibular overjet and posterior cross-bite, was a dominant finding in the group with moderate-severe OI compared to the mildly affected OI group (*P* < 0.050) (Table 4). Frontal open bite and non-occlusion posteriorly were prevalent in both mild and moderate-severe OI.

**Table 4:** Dental Occlusion and Mean Number of Teeth in 69 OI Patients According to OI Type

	OI TYPE	
	Mild (*n* = 52)	Moderate-severe (*n* = 17)
Mean number of natural teeth	26.6	24.2^*^
Min.-max. Number of natural teeth	5–32	14–31
95% CI	25.6–27.6	22.0–26.4
Mandibular overjet	2 (4%)	11(64%)^**^
Increased maxillary overjet (≥ 6 mm)	1 (2%)	0
Deep bite (≥ 5 mm)	2 (4%)	0
Frontal open bite	5 (10%)	5 (29%)
Non-occlusion posterior (in one *or* in both sides)	4 (8%)	3 (18%)
Posterior cross bite (in one *or* in both sides)^a^	7 (15%)	15 (88%)^*^

^*^ *P* < 0.050, Students *t*-test. ^**^ *P* < 0.050, Fischer’s Exact Test

^a^Mild OI: *n* = 48; moderate-severe OI: *n* = 17

## Discussion

The main findings in the present study were that fewer OI patients than expected, independently of the disorder severity, suffered from temporomandibular disorders, had jaw functional limitations, and/or were psychosocially affected. To our knowledge, it is the first time TMD prevalence and jaw function have been investigated in an OI population.

The total number of adult patients with OI in Denmark is estimated to be approximately 500. A major strength of the present study is the relatively large number of patients included, but it is however a weakness due to the risk of selection bias that not all Danish OI patients were enrolled. The ten persons who declined to participate in the investigation of orofacial health declined for logistic and time reasons. To avoid too small study groups, the moderate and the severe OI types were merged prior to data analysis. This grouping is supported by the fact that OI type I is characterized by quantitative deviations in type 1 collagen, whereas type III and IV are characterized by qualitative deviations in type 1 collagen [23, 28, 29]. Too disproportional group sizes could possibly contribute to the lack of significant differences of the conditions studied.

The study is cross-sectional, and all OI types were included. Usually, mild disorder cases are difficult to enroll and often only patients who suffer from the severe types of a disorder are willing to participate in clinical studies. It is a strength in the present study that the whole spectrum of OI patients was enrolled. The missing control group is an obvious limitation of the study, but the knowledge on TMD prevalence and jaw functional limitations, which are available in the literature [15, 21, 30], can serve as a source for comparison.

In general, the participating OI patients were positive to the project and contributive to the data collection process. Nevertheless, a study limitation was that a complete data set was not obtained for all participants, resulting in some missing data. For example, some questions in the RDC/TMD History Questionnaire were left unanswered by the patients. Although professionals were present and ready to assist with the questionnaires and help during examination procedures, health limitations and the voluntary aspect of filling out questionnaires had to be respected. Due to the quite personal character of some questions, it was expected that some participants would leave some questions unanswered resulting in some missing data.

A surprising finding of the study was that temporomandibular pain disorders, i.e., myofascial pain without and with limited opening, arthralgia, and osteoarthritis in the OI population were rare and only found in nine out of 75 (12.0%) OI patients. This proportion is very similar to the one in the general population [15, 21]. In contrast to the painful temporomandibular disorders, physical disabilities and skeletal pain are generally very frequent in all OI types [12]. The overall body expression in many OI patients is considerably different compared to healthy subjects, which can provide an expectation of similar alterations orofacially. The pain and disabilities are apparently mainly due to the common fractures related to OI. Fractures in the orofacial area are uncommon, and this might explain the differences in the occurrence of pain. Limited information is available concerning fractures in the orofacial area; however, fractures of facial bones are expected to be rare compared with load-bearing long bones [31, 32].

Of the disc displacement diagnoses, disc displacement with reduction was the only diagnosis found in this OI population (11 (14.7%) OI patients). Osteoarthrosis was found in six (8.0%) patients. Disc displacement and osteoarthrosis are the most common non-painful TMD disorders. The prevalence of these disorders in the present OI population is similar to the general population [21].

The mean jaw opening capacity was normal in both mild and moderate-severe OI patients, however, significantly more moderate-severe OI patients with reduced jaw opening capacity were found compared to mild OI patients. The functional limitations reported were generally minor, apart from limitations related to chewing hard foods, which, however, did not differ significantly between the mild and the moderate-severe OI patients. It is likely that these findings are related to dental factors as the severe OI patients are characterized by more malocclusions compared to the mild OI patients in which the occlusion are less remarkable. Findings of frontal open bite and non-occlusion posteriorly are prevalent in both mild and moderate-severe OI types (Table 4).

Psychosocial status assessment by the use of the GCPS revealed that 62 out of 75 (82.7%) OI patients did not have TMD pain in the prior six months. The remaining scored grade 1 or 2. Grade 1 and 2 with low disability and low/high pain intensity are considered to be reversible conditions. Interestingly, despite their OI condition, no patients demonstrated the grades 3 or 4, high disability and moderately/severely limiting, levels considered to be irreversible.

Fifty-eight out of 75 (77.3%) OI patients answered the RDC/TMD questions for depression symptoms assessment of which 47 (62.7%) demonstrated no signs of such symptoms. Indications of moderate degree of depressive symptoms were revealed in five (6.7%) OI patients and severe in four (5.3%) patients of which the latter were patients with mild OI. It is a limitation of the present study that about 1/5 of the patients did not fill out the questionnaires correctly leading to missing data. A risk of underestimation of the true level of signs of depression due to the missing answers exists and taken the severity of the disorder into account, a high level of depressive symptoms could have been expected. On the other hand, the findings correspond very well with previous findings describing that OI patients are remarkably resilient and able to adapt to difficult life circumstances [33].

Moderate and severe levels of non-specific physical symptoms (somatization), including pain symptoms, were found in 32 (47.8%) of the OI patients. This finding may be due to OI inherent physical symptoms. Yet, half of the patients in both groups had no signs of somatization, which again supports the perception of the resilient nature of OI patients.

## Conclusions

Patients with moderate-severe OI had significantly more malocclusions and lower mean number of natural teeth than patients with mild OI. The psychosocial status of OI patients was remarkably good considering the severity of this disabling systemic disorder. The bodily pain complaints frequently reported to occur in patients with OI are not largely reflected in the orofacial area as painful temporomandibular disorders.

## Abbreviations

DEP: Depression
DI: Dentinogenesis imperfecta
GCPS: Graded chronic pain scale
HO: Horizontal overjet
MOB: Mandibular overbite
OI: Osteogenesis imperfecta
RDC/TMD: Research diagnostic criteria for temporomandibular disorders
SCL-90-R: Symptom checklist 90-R
SOM: Somatization
TMD: Temporomandibular disorders
